# Mitigation of inductive coupling effects on buried pipelines using gradient control conductors of overhead line configuration and hippopotamus optimization algorithm

**DOI:** 10.1038/s41598-026-40852-5

**Published:** 2026-03-03

**Authors:** Kelthoum Hachani, Bentouati Bachir, Djekidel Rabah, Sid Ahmed Bessedik, Ragab A. El-Sehiemy

**Affiliations:** 1 Renewable Energies and Energy Management Laboratory (LMSEERGE), Engineering Materials, Energy Systems, University of Laghouat, Laghouat, Algeria; 2 LACoSER Laboratory, University Amar Telidji of Laghouat, BP 37G, Ghardaïa Road, 03000 Laghouat, Algeria; 3 Electrical Engineering Department, Faculty of Engineering, Kafrelsheikh University, Kafr El-Shaikh, 33516 Egypt; 4 Széchenyi István University, Egyetem Tér 1, Győr, 9026 Hungary

**Keywords:** Buried metallic pipelines, Electromagnetic interference, Nodal network analysis, EHV overhead power line, Hippopotamus optimization (HO), Energy science and technology, Engineering, Materials science

## Abstract

By electromagnetic perturbation effect, the extra-high voltage (EHV) overhead transmission lines can cause significant induced voltages and currents on buried metallic pipelines located in the immediate vicinity. These voltages can present a source of hazard both for the structural I ntegrity of the metallic pipeline and for the safety of personnel responsible for operation and maintenance. This paper proposes the quasi-static modeling of the electromagnetic interference to which a buried metallic pipeline will be subjected nearby an extra-high voltage (EHV) overhead transmission line, under steady-state operating conditions of the power electrical grid. Using the electrical network analysis method to evaluate the induced voltage levels and its effects on the buried pipeline; also, to propose a mitigation strategy if necessary. The results obtained show that the values of the AC induced voltage on the buried pipeline are significant and exceed the limits defined by the international NACE standard. They can cause a risk of electrocution for intervention personnel and accelerate the process of metal corrosion. Therefore, the gradient control mitigation technique of the conductors and their optimal geometric arrangement of EHV transmission line using Hippopotamus Optimization (HO) algorithm were proposed to reduce AC induced voltages within the permissible safety limits, according to the requirements of the NACE Standard. Finally, it should be noted that the implementation of these mitigation approaches have led to remarkable results in eliminating potential risks.

## Introduction

The buried metallic pipelines for the transport of hydrocarbons, natural gas and chemical products are exposed to many stress factors. The main causes of these damages are the aggressive soil nature, induced currents from electric power transmission lines, and stray currents from railway network lines and other electrical power sources^1^. The underground steel pipelines that are located in the immediate vicinity of high-voltage power transmission lines and railway tracks are subject to electrical interference resulting from alternating current generated by power transport electricity lines, and from stray direct current produced by different conductors of power systems. Under normal operating conditions of the power system, the inductive coupling based on electromagnetic induction is the most important AC interference; it is caused by the magnetic field emitted from extra-high voltage (EHV) power transmission lines, due to alternating currents flowing in electrical conductors^2–7^.

The DC electrical interference has often appeared when a direct current power source is grounded. In the case of a fault in the wiring or electrical insulation, stray current is produced., for any buried metal pipeline located in the medium will represent a path of low resistance conductor for the return electric current. Therefore, it will be fundamentally vulnerable to the effects of this stray current. The usual sources of this type of interference are often electrified railways systems, tramways and subways, welding operations, and HVDC electrical power transmission lines. As a result, these interferences can induce stray currents and voltages circulating in the buried metallic pipelines located in close proximity to the sources that cause these electrical interferences^8–11^. In some cases, these generated induced quantities can reach high levels giving rise to multiple detrimental consequences; the steel corrosion is considered the most common harmful effect of electrical interference. In addition, these values can pose electrical risks include electric shock and electrocution to the safety of the intervention operator (maintenance and repair) and public personnel, they can also threaten the integrity of the pipeline itself and the equipment of the cathodic protection. Corrosion of a metal is resulted from the alteration of its chemical properties caused by a chemical and/or electrochemical reaction with its surrounding environment.^12–15^.

Therefore, in such a situation, a corrective action procedure keeps this induced voltage within an acceptable level. Specialized international regulations for safety reasons have recommended different limit values based on the results and recommendations reported in their studies and scientific research conducted. Among the most important of these international regulations is the American National Association of Corrosion Engineers (NACE) which provide standards that are very well respected around the world and have confirmed that to reduce the potentially dangerous level, the AC induced voltage limit on the metallic pipeline must be set to the safe level of 15 V^16–19^. Indeed, under normal operating conditions, it appears important to evaluate the AC electromagnetic interference caused by power transmission lines on a buried metallic pipeline, in order that the unacceptable threat of corrosion on the metallic pipeline and the safety of the intervention operators should be limited^20–23^.

This paper objective is to propose a quasi-static numerical modeling procedure that able to making it possible to analyze the AC electromagnetic coupling between an EHV overhead power lines. In addition, a buried metallic pipeline located parallelly and in close proximity of this overhead power line, using Faraday’s law of electromagnetic induction and nodal analysis method, as well as the quantification of the effects risk of operating personnel electrocution when touched accidentally the metallic pipeline and AC induced corrosion phenomenon. Thus, the designed mitigation technique decreases the induced voltage on the metallic pipeline below safety level; this is accomplished by placing in the proximity of the pipeline a gradient control conductor attached to the pipeline with decouplers for DC isolation.

Finally, an optimization procedure is performed consisting of selecting the optimal values of the physical parameters of the buried pipeline and the geometric arrangement of the overhead transmission line conductors that provide reduced induced voltage on the buried metallic pipeline. This is achieved using a meta-heuristic approach that has demonstrated both effectiveness and simplicity in solving complex optimization problems involving the maximization of an objective function to meet a specific target. The Hippopotamus Optimization (HO) algorithm is a nature-inspired meta-heuristic that mimics the social and territorial behavior of hippopotamuses, particularly their strategies in foraging and resource protection^37^. HO has recently gained attention for its robust exploration and exploitation capabilities, making it suitable for various engineering optimization problems, including those involving electromagnetic compatibility in electrical systems. In the present work, a program developed in the MATLAB environment (version R2016a) was used to implement the proposed optimization process.

## Inductive coupling mechanism

**Fig. 1:**
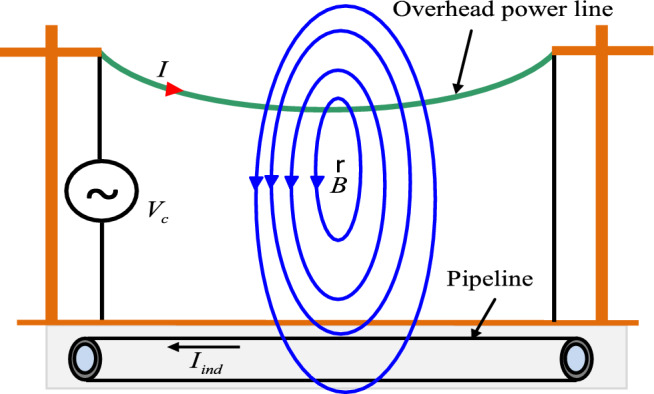
Inductive coupling from EHV power line to metallic buried pipeline.

Electromagnetic coupling is the result of the varying magnetic field generated by the sinusoidal alternating currents flowing in the conductors of the power line, as shown in Fig. 1. The magnetic field generates an induced electromotive force (EMF) at the terminals of the nearby pipeline, causing it to appear an alternating current induced in the pipeline and an induced voltage between the pipeline and the surrounding earth^2–5^. The principle of this coupling is similar to that of an electrical transformer whose conductors and metallic pipeline respectively constitute the primary and secondary windings^2–6^.

## Magnetic induction calculation

**Fig. 2:**
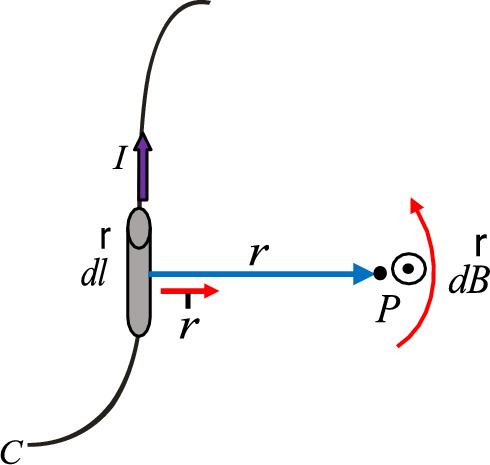
Magnetic induction $\vec{B}$ at point *P* due to a current-carrying element $I{\kern 1pt} d\vec{l}$.

The Biot-Savart law constitutes one of the fundamental laws of magnetostatics. It is an integral equation, which can be approximated as a resultant of magnetic induction contributions from infinitely long conductor segments that are traversed by a distribution of constant electric current. It describes how the resultant magnetic induction B is generated at any point in free space P by a steady electric current; it relates the magnetic induction to its magnitude, direction, length, and proximity to electric current, as shown in Fig. 2. The Biot-Savart law formula can be given as below^27–29^:

1 $$\vec{B} = \frac{{\mu_{0} }}{{4{\kern 1pt} {\kern 1pt} \pi }}\int\limits_{C} {\frac{{I\,d{\kern 1pt} \vec{l} \times \vec{r}}}{{r^{2} }}}$$

Where, $r$ is a vector from the source point to the magnetic induction point; $\vec{r}$ is a unit vector in the direction of $r$; $d{\kern 1pt} \vec{l}$ is a differential element on the circular path $C$ in the direction of the current ; $I$ is the current carrying wire at point source; $l$ is the distance along the current path; $\mu_{0}$ is the permeability of free space.

**Fig. 3:**
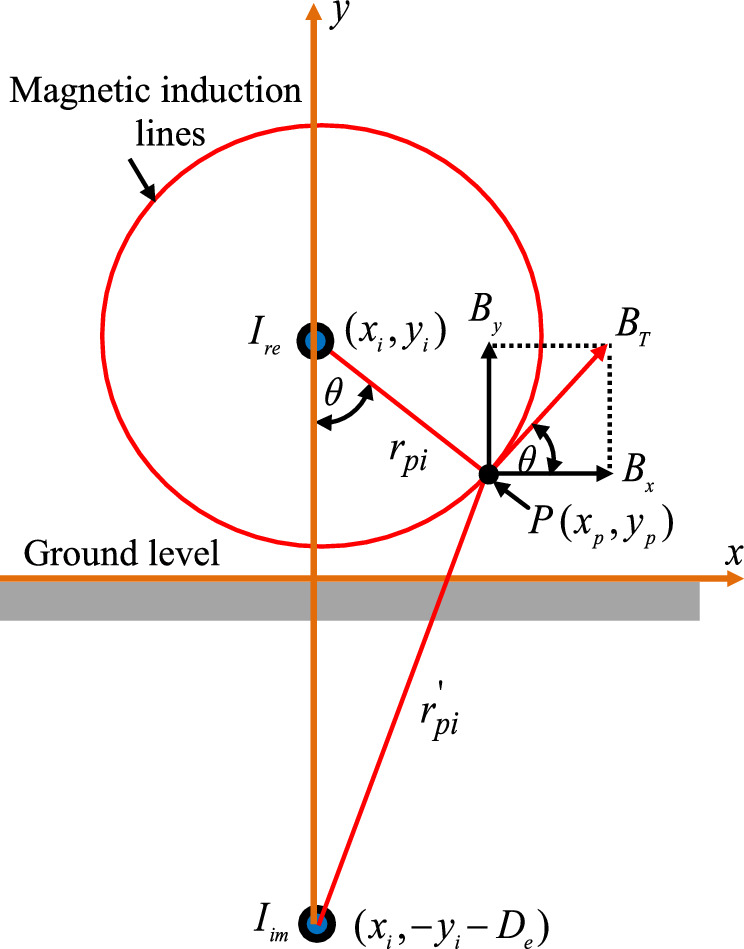
Magnetic induction generated by a single conductor at an observation point.

In the case of a straight conductor, a simple application of the Biot-Savart law makes it possible to determine the magnetic induction B due to the electric current flowing in this conductor at a distance r in a given medium, as shown in Fig. 3, this intensity is represented by^27–29^:

2 $$B = \frac{{\mu_{0} {\kern 1pt} {\kern 1pt} I}}{{2{\kern 1pt} \,\pi \,{\kern 1pt} r}}$$

For the evaluation of the magnetic induction generated in the vicinity of an overhead power line supplied with a balanced three-phase. It is possible to consider in effect the presence of a conductive earth due to the return current induced by the alternating magnetic field that the power line creates it, which is represented by the conductors images located at a depth in the ground equal to their height above the ground plus the complex penetration depth**.** This calculation considers the effect of the induced currents in ground wires and metallic pipeline by the power line currents. The metallic pipeline can be treated as a long conductor with additional loss^29–33^. In a rectangular coordinate plane (x; y), the vertical and horizontal components of the magnetic induction at an observation point P can be expressed as^29–33^:

3 $$\begin{gathered} B_{x} = \frac{{\mu_{0} }}{{2.{\kern 1pt} {\kern 1pt} \pi }}\sum\limits_{i = 1}^{n} {I_{i} } \left[ {\frac{{y - y_{i} }}{{\left( {x - x_{i} } \right)^{2} + {\kern 1pt} {\kern 1pt} \left( {y - y_{i} } \right)^{2} }} - \frac{{y + y_{i} + D_{e} }}{{\sqrt {{\kern 1pt} \left( {x - x_{i} } \right)^{2} + {\kern 1pt} {\kern 1pt} {\kern 1pt} \left( {y + y_{i} + D_{e} } \right)^{2} } }}} \right] \hfill \\ B_{y} = \frac{{\mu_{0} }}{{2.{\kern 1pt} {\kern 1pt} \pi }}\sum\limits_{i = 1}^{n} {I_{i} } \left[ {\frac{{x - x_{i} }}{{\left( {x - x_{i} } \right)^{2} + {\kern 1pt} {\kern 1pt} \left( {y - y_{i} } \right)^{2} }} - \frac{{x - x_{i} }}{{\sqrt {{\kern 1pt} \left( {x - x_{i} } \right)^{2} + {\kern 1pt} {\kern 1pt} {\kern 1pt} \left( {y + y_{i} + D_{e} } \right)^{2} } }}} \right] \hfill \\ \end{gathered}$$

The total magnitude of magnetic induction due to current contributions through all conductors of the power line is expressed by the equation below^29–33^:

4 $$B_{T} = \sqrt {B_{x}^{2} + B_{y}^{2} }$$

The penetration depth of equivalent earth return is given by:

5 $$D_{e} = 658.87\sqrt {\frac{{\rho_{s} }}{f}} {\kern 1pt}$$

Where, $\rho_{s}$ is the soil resistivity; $f$ is the frequency of the source current.

The induced currents in the ground wires and the metal raceway can be determined using the matrix given below^23,33^:

6 $$\left[ {I_{g} } \right]{ = }{\kern 1pt} {\kern 1pt} { - }\left[ {Z_{ii}^{ - 1} } \right] \, \left[ {Z_{ij} } \right]{\kern 1pt} {\kern 1pt} {\kern 1pt} {\kern 1pt} {\kern 1pt} {\kern 1pt} {\kern 1pt} \left[ {I_{c} } \right] \,$$

Where, $Z_{ii}$ are the self-impedances of the (earth wires/pipeline); $Z_{ij}$ are the mutual impedances between phase conductors and (earth wires / pipeline); $I_{c}$ are the currents passing through the phase conductors.

The mutual and self-longitudinal impedances of the conductors can be obtained by the Carson-Clem’s expression, respectively^23,33^:

7 $$Z_{ii} = R_{i} + \frac{{\mu_{0} \,\omega }}{8} + j\frac{{\mu_{0} \,\omega }}{{2{\kern 1pt} \pi }}\left[ {\ln {\kern 1pt} {\kern 1pt} {\kern 1pt} \left( {\frac{{D_{e} }}{{R_{G} }}} \right)} \right]$$

8 $$Z_{ik} = \frac{{\mu_{0} \,\omega }}{8} + j\frac{{\mu_{0} \,\omega }}{{2{\kern 1pt} \pi }}\ln \left( {\frac{{D_{e} }}{{d_{ij} }}} \right) \,$$

Where, $R_{i}$ is the DC resistance of conductor, $R_{G}$ is the geometric mean radius of the conductor; $d_{ij}$ is the distance between the conductor $i$ and the conductor $j$; $\omega$ is the angular frequency.

## Inductive coupling analysis

The amplitude of the induced voltage appearing between the terminals of the metallic pipeline that constitutes a closed circuit, due to the time variation of the electric currents passing through the overhead power line conductors can be calculated using Faraday’s law. This law explains that an induced electromotive force generated in a closed loop placed in a magnetic induction is proportional to the variation over time of the magnetic flux of this magnetic induction, which enters the surface of this circuit. This magnetic flux generated by the varying currents which flows in a surface S is calculated as the integral of the magnetic induction on this surface, as follows^34–38^:

9 $$\phi_{T} = \int\limits_{S} {{\kern 1pt} B_{T} {\kern 1pt} {\kern 1pt} dS}$$

By applying the coordinates of the power line conductors $\left( {x_{i} ,y_{i} } \right)$ and the metallic pipeline $\left( {x_{p} ,y_{p} } \right)$ in order to determine the surface of flux calculation, this magnetic flux can be expressed as follows^34–38^:

10 $$\phi_{T} = - \frac{{\mu_{0} \,\,\,}}{{4\,{\kern 1pt} {\kern 1pt} \pi }}\sum\limits_{1}^{n} {I_{i} } \ln \frac{{\left( {x_{p} - x_{i} } \right)^{2} + \left( {y_{p} + D_{e} + y_{i} } \right)^{2} }}{{\left( {x_{p} - x_{i} } \right)^{2} + \left( {y_{p} - y_{i} } \right)^{2} }}$$

The induced electromotive force on the pipeline can be found^34–38^:

11 $$E_{ind} = - j\,{\kern 1pt} \omega {\kern 1pt} {\kern 1pt} \,\phi_{T}$$

**Fig. 4:**
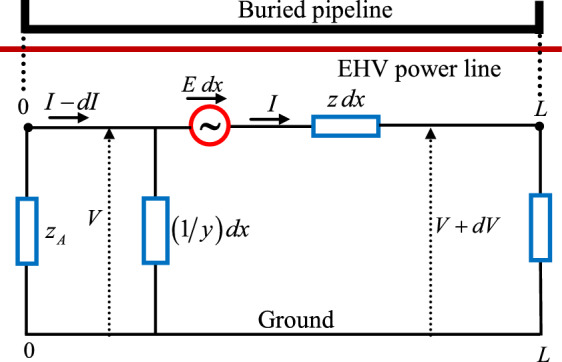
Modeling of equivalent electrical circuit between buried pipeline and ground.

For calculating the induced voltage on buried pipeline, the nodal network analysis is often used which is based on the impedance matrix of the $\pi$ concentrated equivalent circuit type, as shown in Fig. 4. The basic pipeline-earth circuit equations can be written as follows^39–44^:

12 $$V(x) = z{\kern 1pt} {\kern 1pt} dx{\kern 1pt} {\kern 1pt} I(x) + E(x){\kern 1pt} {\kern 1pt} dx - \left[ {V(x){\kern 1pt} {\kern 1pt} + dV(x){\kern 1pt} {\kern 1pt} } \right] = 0$$

13 $$I(x) - dI(x) = y{\kern 1pt} {\kern 1pt} dx{\kern 1pt} {\kern 1pt} {\kern 1pt} V(x) + I(x)$$

The two previous equations given by the relations (12 and 13) are called the fundamental transmission line equations; they are two first order differential equations, with derivatives with respect to the longitudinal coordinate (x). They can be written in the following form^39–44^:

14 $$\frac{dV(x)}{{dx}} + z{\kern 1pt} {\kern 1pt} I(x) - E(x) = 0$$

15 $$\frac{dI(x)}{{dx}} + y{\kern 1pt} {\kern 1pt} V(x) = 0$$

By combining the two complex equations of lines shown in (14 and 15), it is thus possible to obtain two second-order differential equations, as noted below^39–44^:

16 $$\frac{{d^{2} V(x)}}{{dx^{2} }} - y\,z\,{\kern 1pt} V(x) - \frac{dE(x)}{{dx}} = 0$$

17 $$\frac{{d^{2} I(x)}}{{dx^{2} }} - y\,z\,{\kern 1pt} I(x) + y\,E(x) = 0$$

For a pipeline section that continues to run for a several kilometers beyond the both ends A and B of the parallel influence length, which are perpendicular on the EHV power line without earthing.Hence, the general solution of the differential equations represented by (16 and 17) describes the variation of the ground potential along the pipeline section and the current flowing in the pipeline, it is defined by the following exponential functions^39–44^:

18 $$V_{ind} (x) = \frac{{E_{ind} }}{2\,\gamma }\left( {e^{{\gamma \left( {x - L} \right)}} - e^{ - \gamma x} } \right)$$

19 $$I_{ind} (x) = \frac{{E_{ind} }}{{2\,Z_{c} }}\left( {2 - e^{{\gamma \left( {x - L} \right)}} - e^{ - \gamma x} } \right)$$

where, x and L are the positions of the ends of the section of pipeline; $\gamma$ is the propagation constant of the buried pipeline; $Z_{c}$ is the characteristic impedance of the buried pipeline, they are given by^39–44^:

20 $$\gamma = \sqrt {z_{p} y_{p} } {\kern 1pt} {\kern 1pt} {\kern 1pt} {\kern 1pt} {\kern 1pt} {\kern 1pt} {\kern 1pt} {\kern 1pt} and{\kern 1pt} {\kern 1pt} {\kern 1pt} {\kern 1pt} {\kern 1pt} Z_{c} = \sqrt {\frac{{z_{p} }}{{y_{p} }}}$$

The series impedance per unit length with ground return of the pipeline, it is given by ^39–44^:

21 $$z_{p} = \frac{{\sqrt {\rho_{p} \,\mu_{0} \,\mu_{r} \,f} }}{{\left( {3.163} \right){\kern 1pt} {\kern 1pt} r_{p} }} + \pi^{2} \, \times 10^{ - 4} f + {\kern 1pt} {\kern 1pt} j\left[ {\frac{{\sqrt {\rho_{p} \,\mu_{0} \,\mu_{r} \,f} }}{{\left( {3.163} \right){\kern 1pt} {\kern 1pt} r_{p} }}{\kern 1pt} + 4\pi^{2} \times 10^{ - 4} f\log_{e} \left( {\frac{{D_{e} }}{{r_{p} }}} \right)} \right]$$

where, $r_{p}$ is the pipeline’s radius; $\mu_{r}$ is the relative permeability of the pipeline’s metal; $\rho_{p}$ is the pipeline’s resistivity.

The parallel admittance per unit length of the pipeline to the ground is computed using the following formula^39–44^:

22 $$y_{p} = \frac{{\pi D_{p} }}{{\rho_{c} \,\delta_{c} }} + j\,\omega \,\frac{{\varepsilon_{0} \,\varepsilon_{r} \,\pi D_{p} }}{{\delta_{c} }}{\kern 1pt} {\kern 1pt}$$

where, $\rho_{c}$ is the resistivity of the pipeline’s coating; $\varepsilon_{r}$ is the coating’s relative permittivity and $\delta_{c}$ is the coating’s thickness; $D_{p}$ is the pipeline’s diameter.

## Electric shock current

Since the human body is a good conductor of electricity, the electromagnetic interference represented by the AC voltage induced on a pipeline may result in a shock hazard or electrocution hazard to persons accidentally touching this metallic pipeline. In this case, the shock current that would flow through a person’s body is evaluated by combining in series the total impedance of the metallic pipeline, the human body resistance, the body’s contact resistance plus that of the ground resistance of the return path, the current magnitude driven by this electric shock is described by the following expression^44,45^:

23 $${\kern 1pt} I_{sh} = \frac{{V_{ind} }}{{z_{p} + R_{b} + R_{c} }}$$

where, $z_{p}$ is the total impedance of the pipeline; $R_{b}$ is the human’s body resistance; $R_{c}$ is contact resistance of the body.

According to IEEE Std 80, for electrical systems of frequency 50 Hz, from the scientific investigations and research that have been carried out by Charles Dalziel and his colleagues, a mathematical equation has been developed to estimate the risk threshold of the electric shock current which can lead to ventricular fibrillation, the AC admissible body current is supposed to be in accordance with the human body weight and the current contact duration, which can be survived by 99.5 percent of persons, this equation is given by^46–50^:

24 $$I_{th} = \frac{K}{{\sqrt {t_{s} } }}$$

where, $K$ is a factor that depends on the weight of the person body: K = 116 for a human body weighing 70 kg; and K = 157 for a human body weighing 50 kg; $t_{s}$ is the duration of current flow in seconds. The above equation is valid for fault durations between 0.3 and 3 s.

It is clearly noted that the gravity of an electric shock on a human body depends on the quantity of current circulating in the human body and the duration of its passage through this body^46–50^. For a homogenous, the total contact resistance $R_{c}$ is attributed to the contact interfaces of the person; this resistance is estimated by the combination of its contact resistance to the ground and that with the metallic pipeline, it is given by the expression below^46–50^:

25 $$R_{c} = { 3} \times {\kern 1pt} {\kern 1pt} {\kern 1pt} \rho_{s}$$

where $\rho_{s}$ is the electrical soil resistivity.

Regarding the resistance of the human body $R_{b}$, the IEEE Standard 80 recommends a reasonable approximation by a value, which is equal to 1000 Ohm.

In effect, the source of this dangerous electrical shock resides in the indirect contact with the metallic pipeline following to the earth fault, which can lead to dangerous voltage gradients in the ground around the fault site. These are known as the touch and step potentials. The touch voltage arises from the difference between the voltage when a person touches the charged metallic pipeline ,and the voltage at ground surface where the feet’s person are well placed on this ground with a separation distance of 1 m, it can be expressed by the following equation^46–50^:

26 $$V_{touch} = \left( {R_{b} + \frac{{R_{c} }}{2}} \right){\kern 1pt} {\kern 1pt} {\kern 1pt} \frac{K}{{\sqrt {t_{s} } }}$$

The step voltage is represented by the voltage difference between a person’s feet when they are separated with a distance of 1 m, while standing or walking at ground surface, it can be determined by the expression below^46–50^:

27 $$V_{step} = \left( {R_{b} + 2 \times R_{c} } \right){\kern 1pt} {\kern 1pt} \frac{K}{{\sqrt {t_{s} } }}$$

The both equations above concern the maximum tolerable values of the step and touch voltages that must be respected to ensure the safety of persons found in the immediate proximity of the metallic pipeline.

## AC induced corrosion on pipeline

Corrosion is an electrochemical oxidation–reduction reaction with transfer of electrons between a metal and its environment, which leads to a degradation of the metal and its properties, such as hardness or resistance. It is formed when two materials in a structure have different electrical potentials. The potential difference often results from heterogeneity in the metal, in the surrounding medium or the existence of an external electrical source. In the case of iron corrosion, the process consists of two reactions^16,51–54^:

The anodic oxidation half- reaction, with loss of electrons^55–57^:

28 $${\text{Fe }} \to {\mathrm{Fe}}^{{{2} + }} + {\text{2 e}}^{ - }$$

The cathodic reduction half- reaction, with electron gain:

29 $${\mathrm{O}}_{{2}} + {\text{2 H}}_{{2}} {\mathrm{O}} + {\text{4 e}}^{ - } \to {\text{4 OH}}^{ - }$$

The negatively charged hydroxyl ions (OH^−^) produced at the cathode react with the positively charged ferrous ions (Fe^2+^) produced at the anode and form ferrous hydroxide (Fe(OH)_2_), then, the overall redox reaction is:

30 $${\mathrm{Fe}}^{ + 2} + {\text{2 OH}}^{ - } \to {\kern 1pt} {\kern 1pt} {\mathrm{Fe}}{\kern 1pt} ({\mathrm{OH)}}_{2}$$

With more access to Oxygen in the air, the ferrous hydroxide oxidizes to ferric hydroxide; it is formed because of the following reaction^55–57^:

31 $$4{\kern 1pt} {\mathrm{Fe}}{\kern 1pt} ({\mathrm{OH)}}_{2} + {\mathrm{O}}_{{2}} + {\text{2 H}}_{{2}} {\mathrm{O}} \to {\kern 1pt} 4{\kern 1pt} {\mathrm{Fe}}{\kern 1pt} ({\mathrm{OH)}}_{3}$$

The product is the hydrated ferric oxide Fe2O3, commonly called rust. Rust is a thin layer that forms on the surface of metal when it is subjected to a strong oxidizing environment^55–57^.

32 $$4{\kern 1pt} {\mathrm{Fe}}{\kern 1pt} ({\mathrm{OH)}}_{3} + {\kern 1pt} {\kern 1pt} {\kern 1pt} {\mathrm{O}}_{{2}} \to {\kern 1pt} 2{\kern 1pt} {\mathrm{Fe}}_{2} {\kern 1pt} {\mathrm{O}}_{3} {\kern 1pt} {\kern 1pt} .{\kern 1pt} {\kern 1pt} {\kern 1pt} {\text{3 H}}_{{2}} {\mathrm{O}}{\kern 1pt} {\kern 1pt} { + }{\kern 1pt} {\kern 1pt} {\text{2 H}}_{{2}} {\mathrm{O}}$$

The reaction process of AC corrosion is described on the fact that the positively charged ferrous ions produced during the oxidation of iron will interact with the negatively charged hydroxyl ions present during the reduction to form a thin layer deposited on the metal surface. This layer makes it possible to form an insulation barrier (passivation layer); the iron is protected as long as this protective layer of oxide from the corrosion sensitivity covers it. However, the local alteration to passive layer properties over time can induce and accelerate metal corrosion^18,19,58–64^.

For the buried metallic pipeline, corrosion is a phenomenon caused by the induced current exchange between the ground and the metal of the pipeline. This exchange of current depends on the induced voltage that appears at the terminals of the pipeline, it poses a serious threat to pipeline structural integrity, affecting pipeline reliability and lifetime, causing pipeline safety accidents. In the long term, corrosion can lead to a significant loss in the metal of the pipeline of more than 1 mm per year^18,19,58–64^.

The probability of AC induced corrosion can be predicted based on current density levels. According with several international standards relating to corrosion and degradation of materials, associated with a majority of studies and surveys carried out practically by researchers specializing in the AC corrosion field that have dealt the subject of the limit value of AC current density below which AC corrosion does not would not be considered a risk factor. A multiple studies and scientific publications have clearly indicated that corrosion can occur over a current density range of 20 to 30 A/m2^18,19,58–64^.

Assuming uniform and localized corrosion conditions of a homogeneous metallic pipeline material, at a coating circular holiday point, the metallic pipeline has a resistance to remote earth, which can be formulated as follows^65^:

33 $$R_{s} = \frac{{\rho_{s} }}{{2{\kern 1pt} {\kern 1pt} D_{h} }}\left( {1 + \frac{{8{\kern 1pt} {\kern 1pt} {\kern 1pt} \delta_{c} }}{{D_{h} }}} \right)$$

The induced AC current density for a given location depends to the induced voltage on the pipeline, the soil resistivity, and the holiday diameter; it can be calculated according to Ohm’s law as follows^65^:

34 $$J_{ac} = \frac{{V_{ind} }}{{R_{s} {\kern 1pt} {\kern 1pt} {\kern 1pt} S_{h} }} = \left( {\frac{{\frac{{2{\kern 1pt} {\kern 1pt} D_{h}^{2} {\kern 1pt} {\kern 1pt} V_{ind} }}{{\rho_{s} \left( {D_{h} + 8{\kern 1pt} {\kern 1pt} {\kern 1pt} \delta_{c} } \right)}}}}{{\pi \left( {{\raise0.7ex\hbox{${D_{h} }$} \!\mathord{\left/ {\vphantom {{D_{h} } 2}}\right.\kern-0pt} \!\lower0.7ex\hbox{$2$}}} \right)^{2} }}} \right) = \frac{{8{\kern 1pt} {\kern 1pt} V_{ind} }}{{\pi {\kern 1pt} \rho_{s} \left( {D_{h} + 8{\kern 1pt} {\kern 1pt} {\kern 1pt} \delta_{c} } \right)}}$$

where, $J_{ac}$ is the AC current density; $\rho_{s}$ is the soil resistivity; $D_{h}$ is the diameter of the circular holiday; $R_{s}$ is the area spreading resistance of a circular holiday; $S_{h}$ is the surface area of the circular holiday.

The corrosion current can be related directly to the corrosion rate of a material, which is defined as the average rate at which a surface of the metal corrodes uniformly over the entire area that has been exposed to corrosion, it depends on the properties of the metal and the environmental conditions, can be calculated according to Faraday’s law^66–70^:

35 $$CR{\kern 1pt} {\kern 1pt} {\kern 1pt} (cm/y) = \frac{{t \times J_{AC} \times M_{m} }}{{z \times F \times \rho_{m} }}{\kern 1pt} {\kern 1pt}$$

where, M is the atomic weight of the metal (for Fe M = 55.847 g/mol); ρ_m_ is the specific density of metal (for Fe ρ_m_ = 7.85 g/cm3), z is the charge number which indicates the number of electrons exchanged in the dissolution reaction and F is the Faraday constant (F = 96.485 C/mol), for one year $t = 3.16 \times 10^{7} {\kern 1pt} {\kern 1pt} (s)$.

Also in this case, it is important to note that the rate of uniform corrosion can be expressed in terms of metal’s mass loss (weight loss) per unit of surface area and unit of time or decrease in thickness (depth of penetration) per unit of time, it is enough simply to conduct a conversion calculation of units between these quantities.

**Fig. 5:**
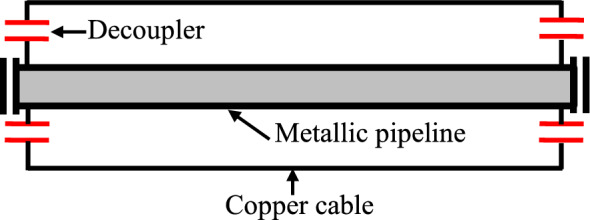
Mitigation technique using gradient control conductor.

In effect, several mitigation techniques could be applied to control and maintain the induced voltage within the limits recommended by the standards on metal pipes exposed to electromagnetic interference. The AC mitigation system allows reducing the induced voltage on the metallic pipeline to a safe level in order to protect personnel and the pipeline from AC induced corrosion and shock potential, when this pipeline runs parallel to an EHV power transmission line. The typical method is to bury a long gradient control conductor of copper cable or a zinc ribbon anode, as illustrated in Fig. 5; it should be installed horizontally and parallel in close proximity to the metallic pipeline and directly connected to this pipeline with decouplers for DC isolation. This mitigation technique is the most common due to its practicality, cost effectiveness and relative cost^71–80^.

The earthing resistance of the copper cable is determined by the resistivity of the soil layer in which the cable is installed and is calculated from the following equation^75–78^:

36 $$R{\kern 1pt}_{c} = \frac{{\rho_{s} }}{{2 \times \pi \times \ell_{c} }}{\kern 1pt} {\kern 1pt} {\kern 1pt} {\kern 1pt} \ln \left( {\frac{{\ell_{c}^{2} }}{{S_{c} \times d_{c} }}} \right)$$

where: $\rho_{s}$ is the soil resistivity; $\ell_{c}$ is the length of copper cable; $S_{c}$ is the burial depth of cable; $d_{c}$ is the diameter of copper cable.

## Developed hippopotamus optimization (HO)

The Hippopotamus Optimization (HO) presents a novel metaheuristic technique inspired by the natural behaviors of hippopotamuses^37^. This approach employs a trinary-phase model to conceptually represent the HO, incorporating evasive maneuvers, defensive strategies against predators, and updates on locations within rivers or ponds, all of which are mathematically formulated. A hippopotamus herd consists of various adult females, calves, multiple adult males, and a dominant male (the herd leader). The iterations of the objective function, which determine the dominant hippopotamus, are used to identify the maximum value for maximization problems and the minimum for minimization problems. Hippopotamuses of the same species typically gather. Dominant males protect the herd and its territory from intruders. Once they mature, dominant males may expel other males from the herd. Subsequently, these ousted males must either attract females or compete with established males within the herd to assert their dominance^37^. The mathematical representation of the location of the dominant male hippopotamus within the herd, situated in a lake or pond, is given in Eq. (37).

37 $$x_{i}^{Mhippo} :x_{i,j}^{Mhippo} = x_{i,j}^{{}} + y_{i} (D_{hippo} - I_{L} x_{i,j} )\,\,\,\,\,\,\,\,for\,i = 1,2,....\left[ \frac{N}{2} \right]\,\,\,\,\,\,\,and\,j = 1,2,....m$$

The position of a male hippopotamus is represented by ${x}_{i}^{Mhippo}$, while the dominant hippopotamus, which holds the optimal solution for the current iteration, is denoted by ${D}_{hippo}$. In Eq. (38), $\overrightarrow{{r}_{5}}$ is a randomly selected value from the interval [0,1]. The terms ${I}_{1}$ and ${I}_{2}$ in Eqs. (37) and (40) are integers ranging from 1 to 2, whereas $\overrightarrow{{r}_{1\dots 4}}$ in Eq. (38) is a randomly generated value between 0 and 1. Additionally, in Eq. (38), ${y}_{1}$ is a random integer between zero and one, and ${m\mathcal{g}}_{i}$ represents the mean values of a few randomly chosen hippopotamuses, each having an equal probability of including the currently evaluated hippopotamus ${x}_{i}$. The parameters ${\varrho }_{1}$ and ${\varrho }_{2}$, appearing in Eq. (38), are random integers that can take values of either zero or one.

38 $$h=\left\{\begin{array}{c}{I}_{2}\times \overrightarrow{{r}_{1}}+\left(\sim {\varrho }_{1}\right)\\ 2\times \overrightarrow{{r}_{2} }-1\\ \overrightarrow{{r}_{3}}\\ {I}_{1}\times \overrightarrow{{r}_{4}}+\left(\sim {\varrho }_{2}\right)\\ \overrightarrow{{r}_{5}}\end{array}\right.$$

39 $$T=exp(-\frac{l}{\Gamma })$$

40 $$x_{i}^{{{\mathcal{F}\mathcal{B}}hippo}} :x_{{i,j}}^{{{\mathcal{F}\mathcal{B}}hippo}} = {\text{ }}\left\{ {\begin{array}{*{20}l} {x_{{i,j}} + h_{1} .(Dhippo - .I_{2} M{\mathcal{G}}_{i} )T} & { > 0.6} \\ \sqsubseteq & { > else} \\ \end{array} } \right.{\text{ }}$$

$$\Xi = \left\{ {\begin{array}{*{20}l} {x_{{i,j}} + h_{2} .(M{\mathcal{G}}_{i} - Dhippo)r_{6} } & { > 0.5} \\ {\ell b_{j} + r_{7} .(u{\mathcalligra{b}}_{j} - \ell {\mathcalligra{b}}_{j} )} & {else} \\ \end{array} } \right.$$

41 $$For\, i=\mathrm{1,2},\dots , \left[\frac{N}{2}\right] and\, j=\mathrm{1,2},...,m$$

The position of female or immature hippopotamuses within a herd is described using Eqs. (40) and (41). Young hippopotamuses may occasionally stray from their mothers due to curiosity. If *T* exceeds 0.6, an immature hippopotamus separates from its mother. When ${r}_{6}$​, a random value within the range [0, 1], is greater than 0.5, the young hippopotamus has moved away from its mother but remains within or near the herd. Otherwise, it has completely separated. The variables ${h}_{1}$​ and ${h}_{2}$​ are randomly selected from five values. The objective function is represented by $\mathcal{F}$. The position update for immature, male, and female hippopotamuses within the herd is determined by Eqs. (42) and (43).

42 $$x_{i} = \left\{ {\begin{array}{*{20}l} {x_{i}^{{{\mathcal{M}}hippo}} {\mathcal{F}}_{i}^{{{\mathcal{M}}hippo}} } & { < {\mathcal{F}}_{i} } \\ {x_{i} } & {else} \\ \end{array} } \right.$$

The use of *h* vectors in the ${I}_{1}$ and ${I}_{2}$ scenarios enhances both the exploration and global search capabilities of the proposed algorithm. This integration strengthens the exploration process, leading to a more effective global search.

43 $$x_{i} = \left\{ {\begin{array}{*{20}l} {x_{i}^{{{\mathcal{F}\mathcal{B}}hippo}} {\mathcal{F}}_{i}^{{{\mathcal{M}}hippo}} } & { < {\mathcal{F}}_{i} } \\ {x_{i} } & {else} \\ \end{array} } \right.$$

The form of the objective function proposed in this optimization algorithm to minimize the induced voltage on the metallic pipeline is represented by^33^:

44 $$OF_{C} = - \sqrt {\left( {V_{\max } - V_{opt} } \right)^{2} }$$

where; $V_{\max \,\,}$ is the initial maximum induced voltage on buried pipeline; $V_{opt\,\,}$ is the new maximum induced voltage after optimization.

The minus sign in the objective function indicates that the optimization problem is a maximization process^33^.

**Fig. 6:**
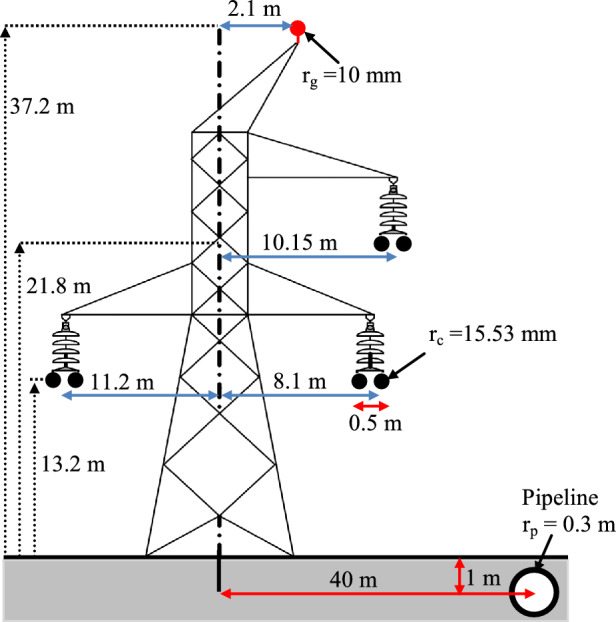
Configuration of single-circuit EHV overhead line with a buried pipeline.

Consider an EHV overhead single circuit transmission line of 400 kV located in Northern Algeria, with a buried metallic pipeline in the immediate vicinity; the arrangement and geometric coordinates of the overhead power line and metallic pipeline are shown in Fig. 6. The metallic pipeline length of exposure to the AC power line is 40 km. The three-phase currents have been assumed under balanced operation with the magnitude of 1800 a; the nominal system frequency is 50 Hz. The earth is assumed to be homogeneous with a resistivity of $50{\kern 1pt} {\kern 1pt} {\kern 1pt} (\Omega {\kern 1pt} .{\kern 1pt} m)$, the AC resistance of the phase (ALMELEC) conductor is $0.0523{\kern 1pt} {\kern 1pt} {\kern 1pt} {\kern 1pt} (\Omega /K{\kern 1pt} m)$, for the earth wire is $0.185{\kern 1pt} {\kern 1pt} {\kern 1pt} {\kern 1pt} \Omega /km$ and $0.5{\kern 1pt} {\kern 1pt} {\kern 1pt} {\kern 1pt} \Omega /km$ for the metallic pipeline. The physical parameters of the buried pipeline are given as follows: the relative permeability of the pipeline $\mu_{r} = 300$; the resistivity of pipeline coating $\rho_{c} = 0.25 \times 10^{7} {\kern 1pt} (\Omega {\kern 1pt} .{\kern 1pt} m)$; the resistivity of pipeline $\rho_{p} = 1.7 \times 10^{ - 7} {\kern 1pt} (\Omega {\kern 1pt} .{\kern 1pt} m)$; the relative permittivity of the pipeline coating $\varepsilon_{r} = 5$; the thickness of the coating $\delta_{c} = 5{\kern 1pt} {\kern 1pt} mm$.

## Results and discussion

The first step consists in estimating the electric current induced in the ground wire in order to take into account its effect in the calculation of the electromagnetic interference within a complex system (EHV power transmission line + buried metallic pipeline), using the relationship earlier mentioned in Eq. (6), which gave the following value $I_{g} = 77{\kern 1pt} {\kern 1pt} {\kern 1pt} e^{{j{\kern 1pt} ( - 18)^{ \circ } }} \left( A \right)$.

The second step corresponds to the quantification of the electromagnetic interference impact, beginning by the magnetic induction evaluation.

**Fig. 7:**
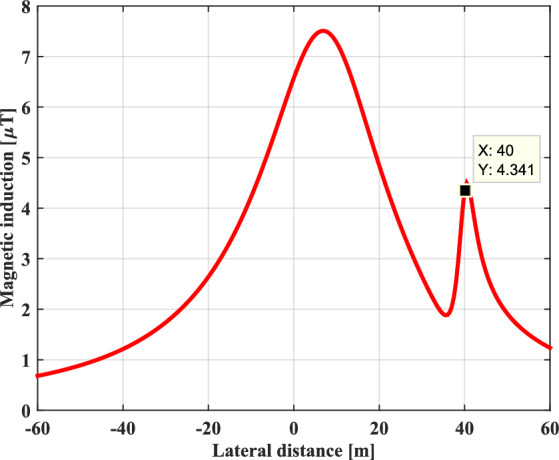
Magnetic induction profile with the presence of a buried metallic pipeline at 1 m.

Figure 7 shows the lateral distribution of the resulting magnetic induction generated by the EHV overhead power line at a height of 1 m above the ground, in the presence of the buried metallic pipeline. It is noted that the existence of a metallic pipeline in the vicinity of an EHV power line disturbs the distribution of the magnetic induction lines. The sudden rise of the magnetic induction at the place where the pipeline is implanted is just due to the induced current effect in the metallic pipeline caused by the electromagnetic coupling, which in turn produces a secondary magnetic induction that will be added to the magnetic induction that was the source of its induction.

**Fig. 8:**
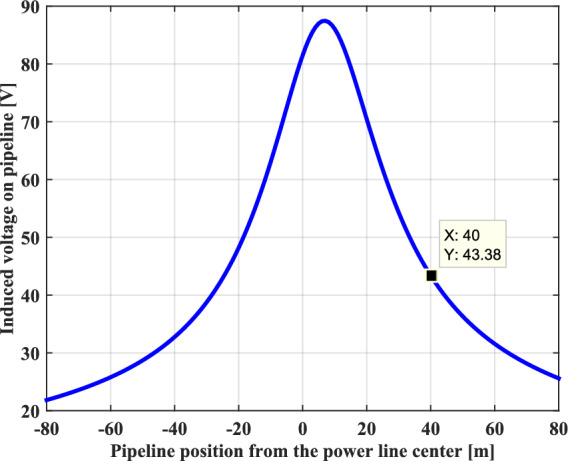
Lateral profile of induced voltage on the buried metallic pipeline.

The profile of the induced voltage on the buried pipeline created by the electromagnetic coupling effect, as a function of different locations of metallic pipeline from the pylon center is shown in Fig. 8. It can be seen that the maximum value is obtained at a distance of 7 m on the side of the of the phase conductors from the pylon center, from this point the induced voltage value decreases progressively with the pipeline location on either side of the right-of-way to reach a very negligible value very far from the pylon. The shape of the induced voltage curve is strongly affected by the geometric arrangement of the phase conductors with respect to the pylon center and to ground level, as well as by the pipeline implantation location and its burial depth.

In our case study, as the pipeline is maintained at a separation distance of 40 m, the obtained value of the induced voltage is equal to 43.38 V. This value is well above the threshold value imposed by the international standard NACE which is 15 V.

**Fig. 9:**
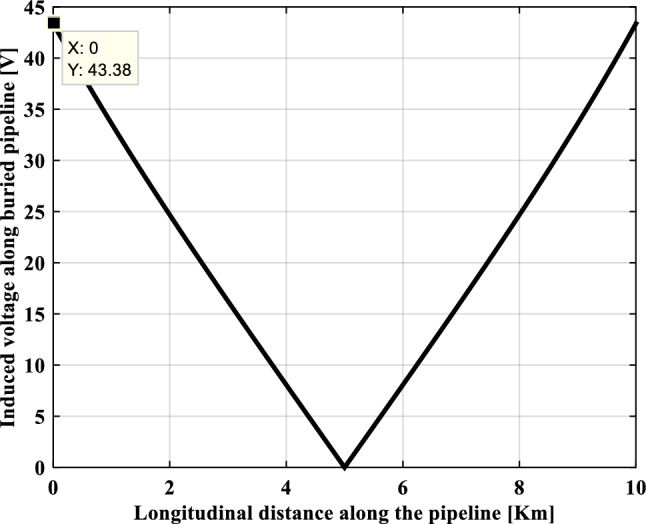
Induced voltage profile along the buried metallic pipeline.

Figure 9 illustrates the profile of the AC induced voltage along the buried metallic pipeline. The longitudinal induced voltage on the pipeline reaches its peak value at the two ends of this pipeline which is of 43.38 V, while this induced voltage is practically zero in the middle of the influence zone distance to the electromagnetic coupling exposure.

**Fig. 10:**
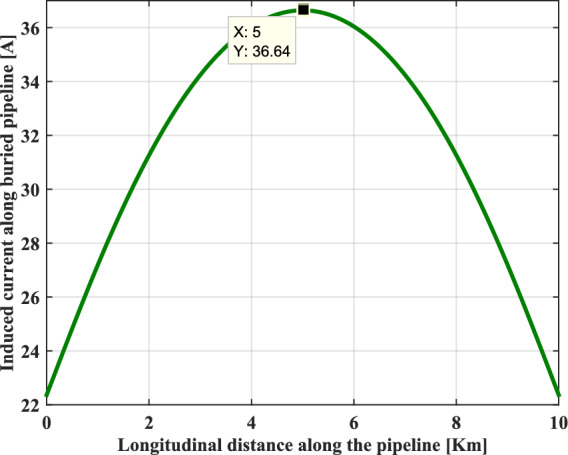
Induced current along the buried pipeline.

The profile of the induced current circulating along the buried metallic pipeline follows an opposite trend to that of the induced voltage. As shown in Fig. 10, it clearly appears that induced current is considerably reduced at both ends of this metallic pipeline, and becomes maximum in the middle of the influence zone distance , where it reaches a peak value of 34.6 A.

**Fig. 11:**
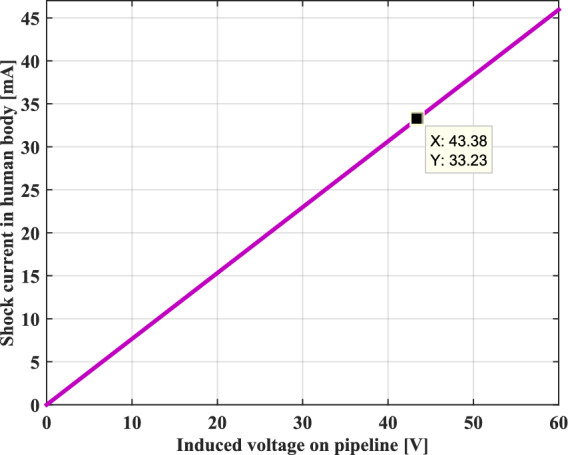
Shock current values passing through the human body.

When a person accidentally touches the metal pipeline under tension, a current can pass through his body and cause an electric shock. Figure 11 depicts the intensity of the shock current passing through a human body as a function of induced voltage across the metallic pipeline under a frequency of 50 Hz. The shock current has a good linear relationship with the applied induced voltage. Accordingly, the higher the induced voltage, the greater the electric shock current.

According to the IEC 61,140 standard, when a human body is traversed by an AC electrical current greater than or equal to a limit value of 30 mA, this person is considered in danger if the current is not interrupted in a short enough time.

In our case study, the current passing through the human body is estimated to be at a value of 33.23 mA, this is a dangerous level of current. Under certain circumstances, an electric current of 20 mA can be lethal, if the person is unable to let go, and the shock time is long enough to cause a deadly shock.

**Fig. 12:**
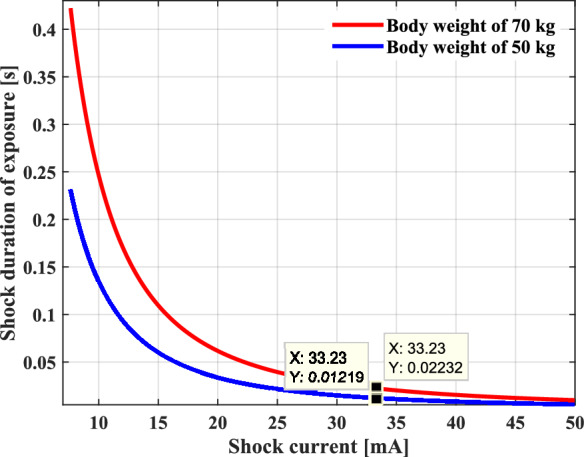
Shock duration of exposure as a function of ventricular fibrillation current (VF).

Figure 12 graphically describes Dalziel’s formula, which gives the relationship between the limiting intensity of the shock current, and the contact time required to produce ventricular fibrillation for different body weights. The body reacts to an increasing current by decreasing the duration time, and in a similar way that it responds to increasing duration as the value of the current decreases, from which it deduces that it takes very short time to induce ventricular fibrillation. For a current threshold of 33.23 mA, it takes about 0.012 s to end the life of a person weighing a maximum of 70 kg and 0.022 s for a person weighing more than 70 kg. It is quite clear that in both cases, the survival time of the person is truly short for a small value of electric current.

**Fig. 13:**
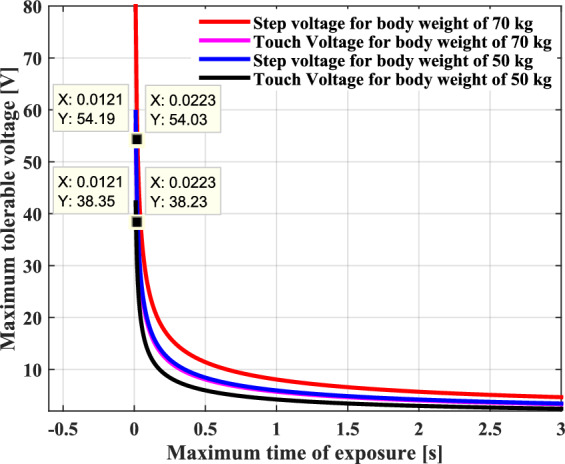
Permissible touch and step voltages as a function of maximum exposure time.

Figure 13 indicates the permissible values of the touch and step voltages as a function of the exposure time to the shock current, these voltages must be respected to guarantee the safety of human body and to avoid ventricular fibrillation during this time. As indicatively shown in this figure, it is evident that as the touch and step voltages increase, the maximum tolerable exposure time decreases, and when these values decrease very rapidly, the exposure time increases. Consequently, the presumed admissible contact and step voltages correspond to a maximum time bearable by the human body and without causing ventricular fibrillation. In the case where the voltage exceeds these allowable values indicated, corrective mitigation measures must be taken.

**Fig. 14:**
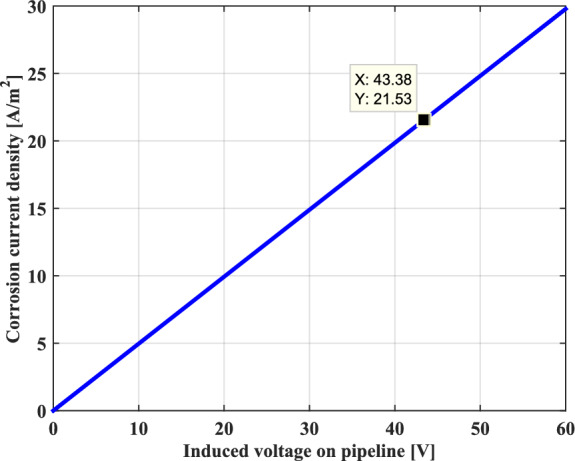
Current density as a function of the induced voltage.

For the probability of the AC corrosion process of pipeline, assuming a coating defect with a diameter of 0.0113 m with a circular holiday having a surface area of 1cm2. Figure 14 presents the AC corrosion current density as a function of the induced voltage in the metallic pipeline for a constant thickness. It can be clearly seen that the AC corrosion current increases linearly with the applied AC induced voltage on the pipeline because of the significant amount of the kinetics of the current exchange oxidation reaction between the metal of the pipeline and the soil located around it. In this case study, the corresponding value of the AC corrosion current density is about 22 A/m2. As a result, according to the above-mentioned limit criterion, the corrosion is expected in the metallic pipeline.

**Fig. 15:**
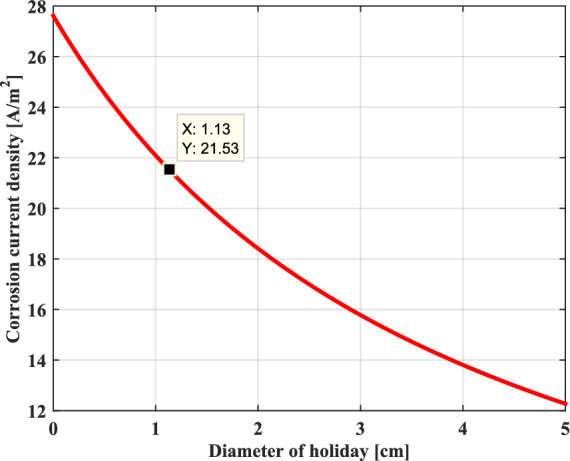
Current density as a function of the holiday diameter.

Figure 15 shows the variation of the AC current corrosion density as a function of the coating defect size; it can be noted that the AC corrosion current decreases inversely proportionally with the diameter of the coating defect. The metallic pipeline which has a coating defect with a small diameter presents a higher risk of corrosion due to significant AC corrosion current. Increasing the size of coating defect reduces the risk of corrosion..

**Fig. 16.**
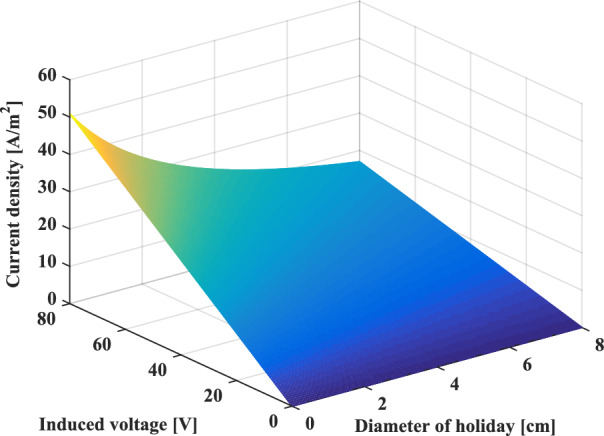
3D Three-dimensional profile of AC corrosion current density.

Figure 16 describes the three-dimensional (3D) variation of the current AC density as a function of the induced voltage and the size of the coating defect. From the figure, the potential risk of corrosion increases with the rise in the induced voltage imposed on the metallic pipeline. On the other hand, this risk decreases when the coating defect size increases.

**Fig. 17:**
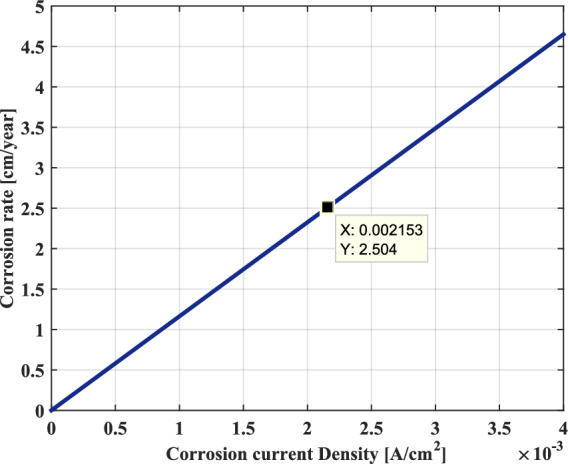
Corrosion rate as a function of AC corrosion current density.

Assuming, the condition of uniform corrosion of a homogeneous material, Fig. 17 represents the corrosion rate of the metal expressed in cm/year as a function of the AC corrosion current density. As the oxidation rate is controlled by the charge transfer process (electrons) at the metal/solution interface. It can be seen in this figure that the corrosion rate progresses linearly with the current density, which runs through the corroded metal. Indeed, for an intensity given of AC corrosion current, the corrosion rate will be greater the smaller the surface of the metallic material or the higher the generated induced voltage, which leads to an increased risk of steel corrosion.

**Fig. 18:**
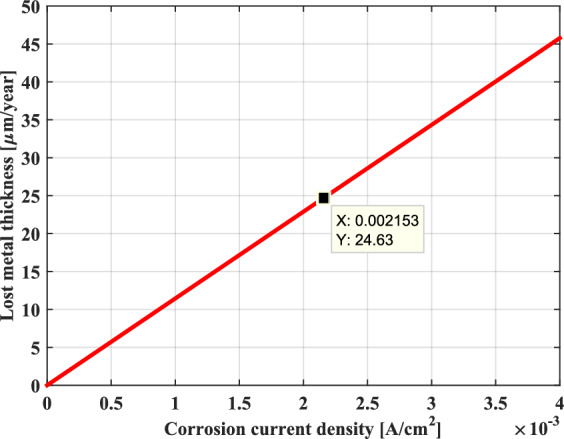
Decrease in thickness as a function of AC corrosion current density.

**Fig. 19:**
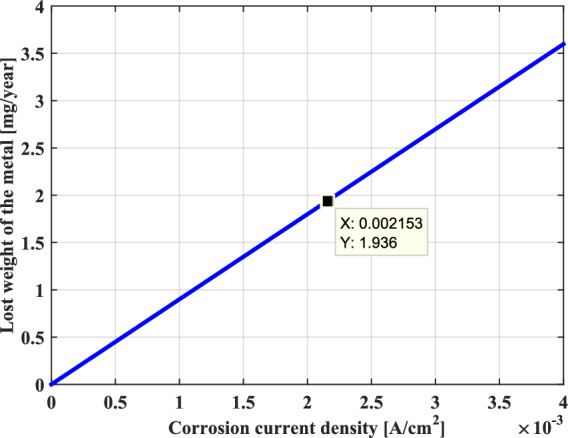
Metal’s mass loss as a function of AC corrosion current density.

Furthermore, the corrosion rate can also be expressed in terms of the decrease of the metal thickness per unit time or the metal’s mass loss per unit time. As shown in Figs. 18 and 19, it can be seen that the behavior represented on these two graphs is similar and that these terms increase linearly with the AC corrosion current density.

Consequently, it can very well conclude that the uniform corrosion behavior of the metal is characterized by a corrosion rate that is proportional to the AC corrosion current density. In effect, this corrosion can better evaluate with the weight loss and the thickness reduction of metal, which increase proportionately with the rise in the AC corrosion current.

The last step is to install a preventive measure for the protection of personnel conducting repair and maintenance work against electric shocks, also to prevent corrosion of buried metal pipelines, by reducing the induced voltage on the buried metallic pipeline to acceptable limits in order to avoid all potential risks.

The international standard NACE recommends that to avoid the level of dangerous potential, the AC induced voltage on the pipeline must reach to a target value, which must be lower than 15 V.

in this case study, to reduce the potential of the pipeline to the safe limit, an attenuation system using a gradient control copper cable is installed parallel and close to the section of the pipeline and regularly connected to it .

Under steady-state conditions, the Gradient Control Conductor provides the most complete attenuation possible because it prevents the pipeline coating from being electrically overloaded and allows contact voltage to be uniformly minimized throughout the attenuated section of the pipeline.

**Fig. 20:**
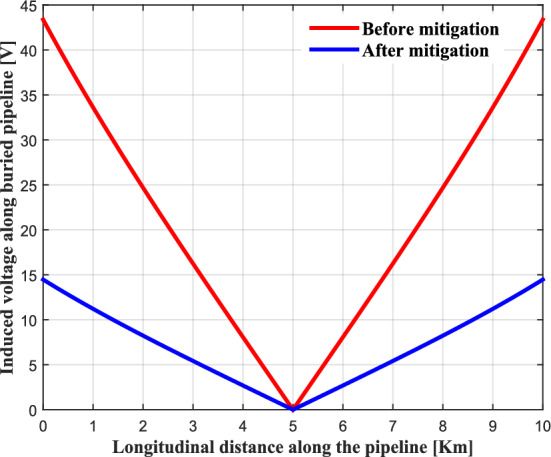
Induced voltage profile along the pipeline before and after mitigation system.

Figure 20 shows the values of the induced voltage along the pipeline before and after the installation of the mitigation system. This figure shows the maximum value of the induced voltage has been set at a limit close to 15 V with respect to the local earth. This threshold motivates by safety considerations (risks of electrocution and corrosion). The performance of the proposed mitigation technique is quite effective and reliable. It allows significantly to reduce the peak value of the induced voltage appearing at the terminals of the pipeline.

**Fig. 21:**
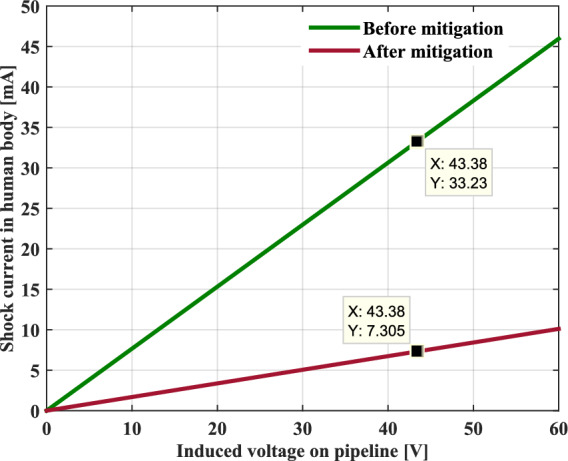
Shock current in human body before and after mitigation system.

**Fig. 22:**
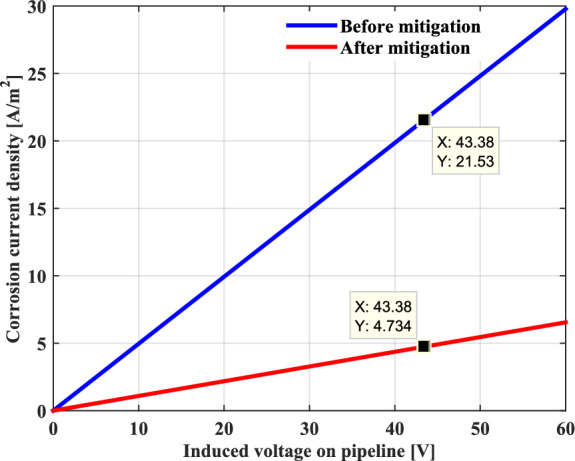
AC corrosion current density before and after mitigation system.

Figures 21 and 22 present the simulation results obtained for the electric shock current through the human body and the corrosion current density before and after the installation of the proposed gradient control conductor, respectively. It can be seen quite clearly that these two quantities of risk on the buried pipeline have been reduced to low values, which are well below the safe limits specified in the NACE International Standard.

However, the induced voltage on buried pipeline can also be minimized using optimization algorithms by determining the optimal arrangement of overhead power line conductors that achieves the lowest induced voltage based on an objective function maximization process. The Hippopotamus Optimization (HO) algorithm exhibits strong power in accuracy and efficiency and can be adopted as a reliable method to overcome the optimization problem in question.

**Fig. 23:**
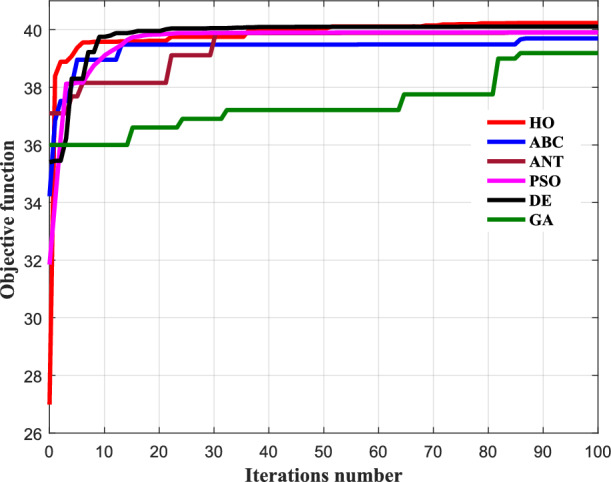
Convergence of objective function of all algorithms with number of iterations.

Figure 23 illustrates the variation of the objective function value as a function of the number of iterations. It highlights the optimization process of the algorithm, which consists of maximizing the objective function specified in Eq. (40) above. Figure 23 shows the convergence characteristics of the objective function for the different optimization algorithms. All methods exhibit rapid improvement during the initial iterations due to effective exploration of the search space. However, clear differences appear in convergence speed and final objective function values. HO algorithm converges faster and reaches a higher final objective function value compared to PSO, GA^81^, ANT^82^, DE, and ABC. This indicates a stronger capability to avoid local optima and to identify a more effective conductor configuration for reducing the induced voltage on the buried pipeline. Moreover, HO demonstrates a smooth and stable convergence behavior with minimal oscillations, reflecting its robustness and reliability.

Overall, Fig. 23 confirms the superior performance of the HO algorithm in terms of convergence efficiency, solution quality, and stability for the proposed electromagnetic interference mitigation problem.

Table 1 compares the performance of HO with several metaheuristic algorithms in terms of objective function value. The HO algorithm achieves the highest value (40.24), indicating a more effective reduction of the induced voltage on the buried pipeline. Other methods, including ANT and DE, show competitive but slightly lower performance, while ABC, PSO, and GA yield lower objective values. These results confirm the effectiveness of HO for the proposed optimization problem.

**Table 1:** Performance comparison of metaheuristic algorithms for induced voltage mitigation.

Optimization algorithms	Setting parameters	Objective function value
HO	nPop = 20	40.24
ABC	nPop = 20; L = 10	39.69
ANT	nPop = 20; alpha = 0.04; beta = 0.01; roh = 0.03	40.07
PSO	nPop = 20; wmax = 1.5; % wmin = 0.2; c1 = 2; c2 = 2	39.90
DE	nPop = 20; beta_min = −1.5; beta_max = 1.5; part = 0.95	40.01
GA	nPop = 50; pc = 0.7; gamma = 0.4; pm = 0.3; mu = 0.1	39.19

**Fig. 24:**
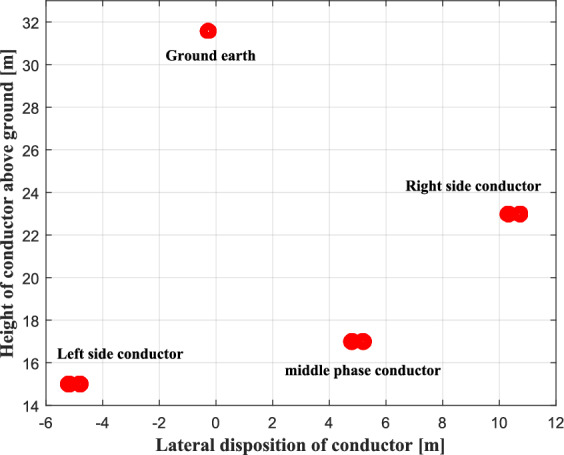
Optimal geometric arrangement of the EHV overhead transmission line conductors.

The simulation results of the optimal phase conductor arrangement for an overhead transmission line, obtained using the HO optimization algorithm, are presented in a two-dimensional graph. As shown in Fig. 24, it can be clearly observed that the optimal phase conductor arrangement is an equilateral triangular arrangement, and the ground wire is positioned with the central phase conductor in the plane of symmetry. This demonstrates that the triangular phase conductor configuration results in a significant reduction in the magnetic field and therefore a reduction in the induced voltage.

**Fig. 25:**
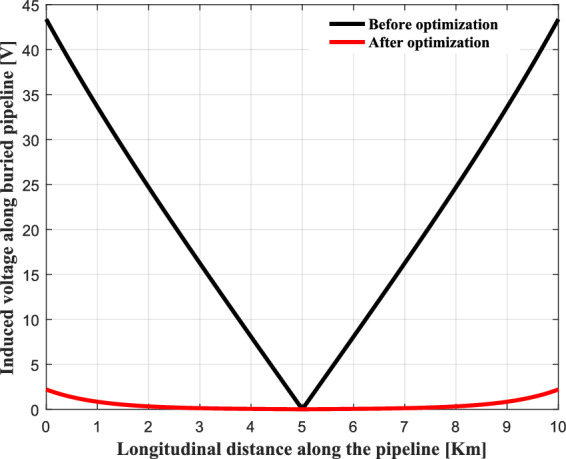
Induced voltage profile along the pipeline before and after optimization process.

Figure 25 illustrates the induced voltage in the metallic pipeline, resulting from the optimal location of overhead power line conductors. A significant decrease in this voltage is observed, with a maximum value of 2.35 V. This is attributed to the optimized distribution of the phase conductors, which are sufficiently spaced horizontally and positioned at different heights above the ground, in accordance with safety requirements. This arrangement creates a geometric asymmetry, similar to a flattened triangle, allowing for adjustment of the directions of the magnetic field vectors and improving their partial cancellation. Consequently, a reduction in the magnetic field strength and in the induced voltage since it is directly proportional to the value of the total magnetic field. As for the ground earth, its optimized positioning above the phase conductors, at the center of the asymmetry, effectively protects the horizontal span of the phase conductors against lightning strikes.

The results of the optimization of the adopted algorithm for the horizontal position and height of the electric line conductors are summarized in Table 2.

**Table 2:** Optimized geometric location of overhead power line conductors.

	Lateral position relative to symmetry point (m)	Height above ground (m)
Left conductor	− 5	15
Middle conductor	+ 5	17
Right conductor	+ 10.5	23
Ground earth	− 0.25	31.6

When using a single ground earth, its usual location is at the top of the configuration horizontally between the phase conductors. This location ensures adequate protection. For optimal and reliable protection, it is recommended to add a second ground earth, to fully cover the horizontal span of the phase conductors and prevent accidents, especially in areas with high storm activity. As a suggestion, this point could be addressed in a future study.

## Conclusion

In this paper, a quasi-static modeling based on Faraday’s law and nodal network analysis is carried out for the electromagnetic coupling evaluation between a buried metallic pipeline and an EHV overhead power line under normal operating conditions of the electrical power grid. From the results, it is very evident that the presence of a metallic pipeline in proximity of an overhead power line causes a significant distortion in magnetic induction lines at pipeline’s surface due to the induced current generated by electromagnetic induction effect. The AC induced voltage in metallic pipeline as a function of its position relative to the pylon center reaches a maximum value for a critical position of its laying which is close to the side conductor, then decreases rapidly with increasing the pipeline’s pose position in both sides of this critical point. The longitudinal induced voltage in the metallic pipeline is higher at its both ends and zero in the middle of its length. The longitudinal current value is maximum at the middle of its length and is reduced at its two ends. Consequently, the maximum induced voltage obtained is significant and exceeds the threshold value established by the NACE international standard, it can cause an electric shock for the operating personnel who accidentally touch the pipeline, it can also lead to a corrosion problem through any pipeline coating defect. Finally, it is suggested to implement a mitigation procedure consisting of installing a gradient control conductor in the immediate vicinity of the buried pipeline, in order to reduce the induced voltage to the safe limit as indicated in the NACE international standard to eliminate the damage associated with risks. It is observed that this mitigation system is considered appropriate and highly effective in reducing the electromagnetic coupling effect to a safe level in terms of personnel safety and pipeline integrity. To significantly improve the induced voltage reduction, the position of the overhead line phase conductors was also considered by performing an optimization procedure based on an efficient and reliable approach of Hippopotamus optimization. The optimization results showed that arranging the phase conductors in a triangular shape leads to a significant reduction in the maximum induced voltage value. This is due to the cancellation of the magnetic field produced between the phase conductors due to their being positioned at equal distances from each other. Finally, regarding the prospects of this research, the scope of this study can be broadened to take into account faults that could appear during transient operating regimes of electrical networks such as switching, short circuits and lightning discharge, where the effect of inductive and conductive coupling becomes more serious and more sensitive.

## Data Availability

The data required are available upon acceptance via the first and corresponding authors.
